# Plasmon Engineering in Intercalated 2H-TaS_2_

**DOI:** 10.1021/acs.nanolett.6c01548

**Published:** 2026-07-10

**Authors:** Luigi Camerano, Laura Martella, Lorenzo Battaglia, Federico Giannessi, Filippo Camilli, Luca Lozzi, Polina M. Sheverdyaeva, Paolo Moras, Luca Ottaviano, Gianni Profeta, Federico Bisti

**Affiliations:** † Department of Physical and Chemical Sciences, 9303 University of L’Aquila, Via Vetoio, 67100 L’Aquila, Italy; ‡ CNR-SPIN L’Aquila, Via Vetoio, 67100 L’Aquila, Italy; § CNR-Istituto di Struttura della Materia (CNR-ISM), Strada Statale 14, km 163.5, 34149 Trieste, Italy

**Keywords:** plasmons, TMD, intercalation, core-levels, electron energy loss, photoemission

## Abstract

Plasmons in low-dimensional materials provide a powerful platform for nanoscale control of light–matter interactions, yet strategies to tailor their coherence and dissipation remain limited. Here, we demonstrate that transition-metal intercalation offers a fundamentally distinct route to engineer plasmonic response in layered materials. By combining high-resolution core-level photoemission spectroscopy with first-principles calculations, we show that Fe and Co intercalation in 2H-TaS_2_ does not act as conventional electron doping but reshapes the low-energy electronic structure through orbital hybridization and structural reconstruction. This process introduces a dense continuum of low-energy states that ultimately suppresses the plasmon mode. First-principle calculations of the energy-loss function reveal a transition from a well-defined collective excitation to an overdamped response. Our results establish intercalation as a chemically controlled pathway to tune plasmon losses and dielectric response in quantum van der Waals materials, providing a new design principle for plasmonic and optoelectronic functionalities at the nanoscale.

## Graphical abstract

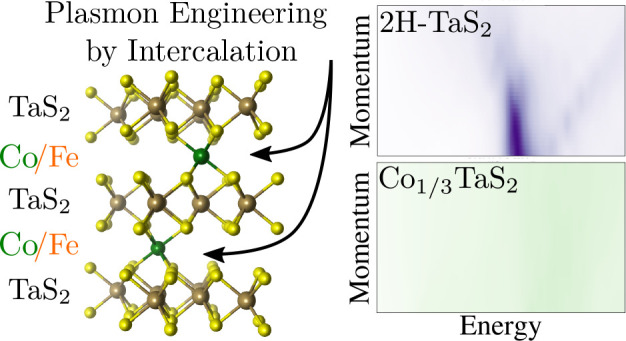

Collective electronic excitations encode fundamental properties of quantum materials, reflecting the intricate interplay between electronic structure, dimensionality, and many-body interactions.^1,2^ Among these, plasmons, coherent oscillations of the charge density, are uniquely sensitive to low-energy electronic states and dynamical screening.^3–5^ Their tunability under external stimuli has positioned plasmons at the forefront of condensed-matter physics and nanophotonics, with broad implications for optoelectronics, sensing, and quantum technologies.^6–10^ In low-dimensional materials, where Coulomb interactions and band topology are enhanced, plasmon modes are extremely sensitive to subtle changes in crystal structure and orbital character, offering a direct window into emergent electronic phenomena.^11–14^ Layered transition-metal dichalcogenides (TMDs) are an ideal platform to investigate and control these collective excitations,^15–18^ thanks to their van der Waals structure, which allows chemical manipulation via gating,^19^ substitutional doping,^20^ and intercalation,^21^ accessing a wide range of correlated and topological phases. Among these, transition-metal intercalation is particularly powerful, enabling long-range magnetic order,^22–24^ spin-textured phases,^25–28^ and tunable electronic properties.^29,30^ Yet, despite this versatility, how intercalation modifies plasmon excitations and the overall dynamical electronic response remains largely unexplored. Here, we address this challenge by studying plasmon excitations in 2H-TaS_2_, a prototypical layered metal with intertwined metallicity, charge-density-wave (CDW) order, and hyperbolic light dispersion.^31–35^ In this system, electron-energy loss spectroscopy (EELS) has revealed a plasmon mode with negative momentum dispersion,^15^ reflecting the low-energy electronic structure governed by isolated metallic bands, rather than CDW induced modification of the electronic structure as reported in refs. 18,35–37 Such collective excitations leave measurable fingerprints in core-level spectra, arising from both many-body screening and inelastic loss processes.^38–40^ Early Ta-4*f* measurements indeed revealed complex, multicomponent lineshapes,^41–43^ suggesting a rich interplay between electronic structure and collective modes. However, only a quantitative line shape description can directly connect these spectral features to plasmon dispersion and its evolution upon intercalation. By combining high-resolution synchrotron-radiation-based core-level spectroscopy and laboratory X-ray photoelectron spectroscopy (XPS) with first-principles calculations, we investigate plasmon excitations in 2H-TaS_2_ and its Fe and Co-intercalated compounds, namely the Ising ferromagnet Fe_1/3_TaS_2_,^44–47^ and the noncoplanar antiferromagnet Co_1/3_TaS_2_.^48–50^ By carefully modeling the core-level line shape and explicitly accounting for low-energy excitations arising from the electronic density of states (DoS) calculated from first-principles Density Functional Theory (DFT), we show that plasmonic features strongly shape the spectra of 2H-TaS_2_. Upon intercalation, these low-energy excitations are strongly suppressed in both Fe_1/3_TaS_2_ and Co_1/3_TaS_2_, reflecting the emergence of additional damping channels driven by cooperative structural reconstruction and orbital hybridization rather than simple electron doping. These results demonstrate that transition-metal intercalation provides a chemically controlled route to engineer plasmon losses and, more broadly, the collective electronic response in layered materials.

Bulk 2H-TaS_2_ is formed by stacked 1H-TaS_2_ monolayers (MLs) in an ABA sequence related by a glide-mirror symmetry. The 1H-TaS_2_ ML consists of a hexagonal lattice of Ta atoms in trigonal prismatic coordination with the chalcogen.^51^ Fe and Co intercalation in the interlayer spacing of metallic 2H-TaS_2_ is ordered into a $\sqrt{3}\times \sqrt{3}R30^{{}^{\circ}}$ reconstruction and induce magnetism in the system as shown in Figure 1a-b. Our Low Energy Electron Diffraction (LEED) patterns is accordingly composed by the 1 × 1 spots and the additional periodicity compatible with $\sqrt{3}\times \sqrt{3}R30^{{}^{\circ}}$ reconstruction (see the orange and green reciprocal space lattice vectors for Fe and Co, respectively in Figure 1c-d).

**Figure 1:**
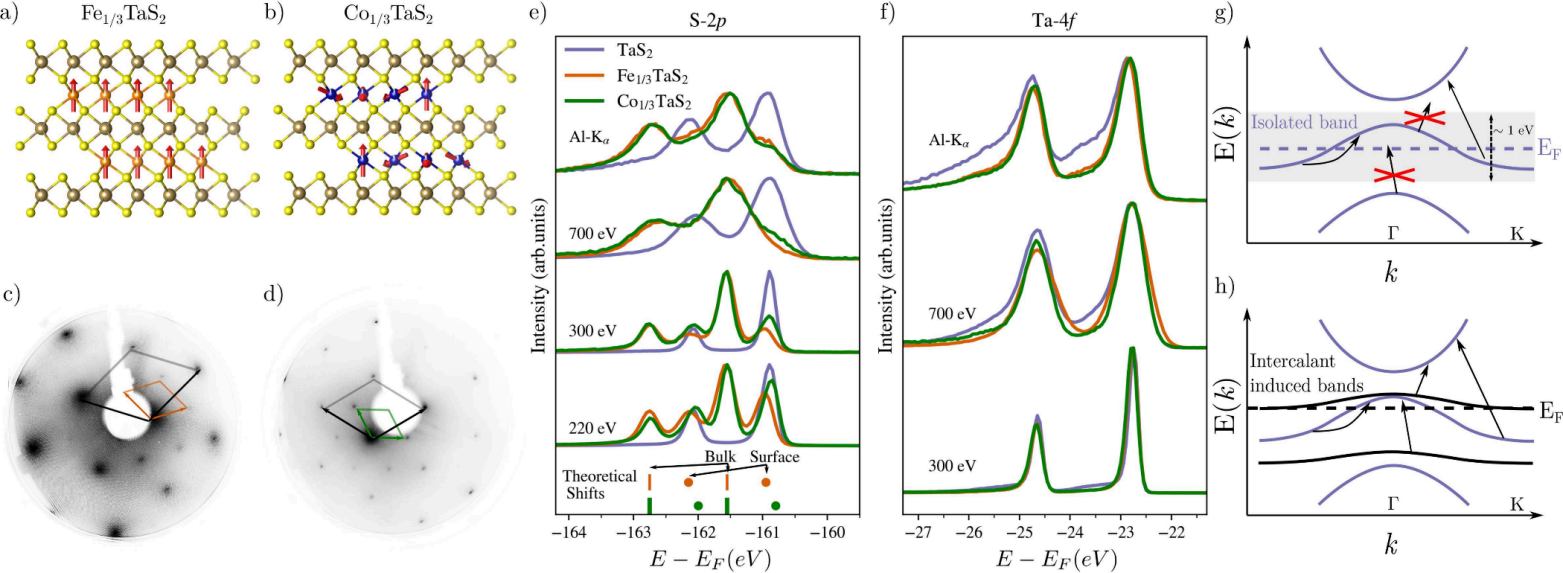
a) Crystal structure of ferromagnetic Fe_1/3_TaS_2_ and b) noncollinear triple-Q state of Co_1/3_TaS_2_ with *c*-axis aligned along the *z*-direction. c) and d) LEED spots highlighting the $\sqrt{3}\times \sqrt{3}R30^{{}^{\circ}}$ reconstruction in both Fe_1/3_TaS_2_ and Co_1/3_TaS_2_, respectively. e) S-2*p* core-level line shape for different photon energies and different compounds. Photon-dependent spectra were acquired at *T* = 20 K at VUV beamline (Elettra) while Al–K_α_ spectra (hν = 1486.7 eV) were acquired at room temperature. In the bottom of the figure we report theoretical bulk-surface core-level shifts calculated from initial state theory (bulk with lines, surface with dots). f) Photon-energy dependent Ta-4*f* core-level for 2H-TaS_2_, Fe_1/3_TaS_2_ and Co_1/3_TaS_2_ acquired at hν = 300 eV and hν = 700 eV. Sketch of the interband transition facilitated by intercalant induced band from 2H-TaS_2_ g) into Fe/Co_1/3_TaS_2_ h). The black arrows indicate allowed electronic transitions from occupied to unoccupied states, while the red crosses mark forbidden transitions.

Beyond structural reconstruction, intercalation strongly alters the electronic properties of 2H-TaS_2_. Figure 1e shows the photon-energy–dependent S-2*p* core-level spectra aligned to the Fermi level as determined from the valence band (see SI for the details and refs 24,41,48,50,52–70 therein, Figures S1–S2). Using Al–K_α_ (hν = 1486.7 eV), pristine 2H-TaS_2_ exhibits a single S-2*p* main component centered at ∼ – 160.9 eV, with no additional features emerging upon varying the photon energy. In contrast, Fe_1/3_TaS_2_ and Co_1/3_TaS_2_ show a chemical shift of the main sulfur peak relative to the pristine compound, together with the appearance of a low-binding-energy shoulder that evolves into a well-resolved peak at lower photon energies. The intensity of this additional component increases with decreasing photon energy, consistent with a surface contribution associated with a sulfur-terminated layer (see SI for angle-dependent XPS, in particular Figures S5–S6). Notably, the binding energy of the S-2*p* peak in pristine 2H-TaS_2_ closely matches that of this surface component in the intercalated systems. Our first-principles slab calculations within the initial-state approximation reproduce this bulk–surface splitting (0.6 eV for Fe_1/3_TaS_2_ and 0.75 eV for Co_1/3_TaS_2_, see the bottom of Figure 1e), confirming that intercalation delocalizes charge around S atoms, reducing its nucleus electronic screening during photoemission process. The combined bulk shift and surface feature provide a clear spectroscopic fingerprint of intercalant–sulfur bonding. These modifications of the S chemical environment likely influence magnetic interactions, naturally explaining deviations from a purely RKKY-mediated exchange, where magnetic coupling arises from the interplay of RKKY and chalcogen-mediated superexchange.^71,72^

In Figure 1f we report the photon-energy-dependent Ta-4*f* core-level spectra, aligned to the Fermi level as determined from the valence band (see SI for the details). We first observe that, despite the doping of the valence band induced by intercalation as reported in refs,^24,73–75^ the Ta-4*f* core-level binding energy does not exhibit any appreciable shift among the three compounds. This indicates that the changes in carrier concentration do not significantly modify the local electrostatic potential at the Ta site, consistent with efficient metallic screening (this is in contrast with prior studies on these compounds^76^). Beyond the two sharp peaks corresponding to Ta-4*f*_7/2_ and Ta-4*f*_5/2_, we observe, particularly in 2H-TaS_2_, a broad spectral feature in the high-binding-energy tail. Its intensity increases at higher photon energy and it gets broader in the intercalated compounds. The enhancement of this feature at higher photon energies points to its origin as an extrinsic loss process, arising from inelastic scattering of the photoelectron during its propagation to the surface. At higher photon energies, the emitted photoelectrons possess greater kinetic energy and can escape from deeper layers of the sample, increasing the effective probing depth. As a consequence, a larger fraction of the detected electrons undergoes one or more inelastic scattering events along the escape path while still retaining sufficient energy to leave the sample, thereby enhancing the extrinsic loss contribution to the spectrum.^63^ Such a feature can also be traced in the S-2*p* core level, although it is less pronounced (see Figure S7 in SI). Moreover, EELS measurements on pristine 2H-TaS_2_ confirm the presence of a plasmon resonance at ∼1 eV,^15^ and previous analyses of the core-level spectra required additional components to accurately reproduce the line shape.^41,42^ The origin of this plasmon excitation in 2H-TaS_2_ can be traced to the isolated metallic band characteristic of 2H metallic phases of TMDs,^18,35–37^ as illustrated in the sketch in Figure 1g. The observed suppression of this signal in core-level spectroscopy can be understood as a consequence of the additional electronic states introduced by the intercalated transition metal as recently measured in angle-resolved photoemission spectroscopy (ARPES),^24,74,75^ which open new low-energy absorption channels (see sketch in Figure 1h).

To support this interpretation, we calculated the density of states (DoS), shown in Figure 2a. Indeed, we find that Fe intercalation induce *n*-doping the Ta- $d^{z^{2}}$ bands at the Fermi level^24,34^ and introduces additional low-energy states arising from Fe-*d* and Ta-*d* hybridization. In contrast, Co intercalation produces a stronger modification of the pristine DoS. This trend is consistent with ARPES measurements,^24,34,73^ which also report an increased density of states near *E*_*F*_ in Co-intercalated TaS_2_ with respect to Fe intercalated counterpart. Moreover, the calculation of the DoS allows us to quantitatively isolate the extrinsic loss contribution by modeling the core-level line shape from first-principle calculation. Indeed, modifications of the DoS near the Fermi level in metals affect the shape of the many-electron singularity observed in X-ray photoemission.^77,78^ The widely used Doniach–Šunjić (DS) asymmetric line shape can be generalized to include the influence of the electronic DoS by expressing the core-level intensity in the time domain:^79–82^

1 $$\begin{array}{ll} I\left(E\right) & ∼\int_{-\infty }^{+\infty }e^{i\left(E-E_{0}\right)t-\lambda \left|t\right|-\sigma^{2}t^{2}/2-g\left(it\right)}dt\\ g\left(\tau \right) & =\alpha \int_{0}^{\infty }\frac{\rho \left(E\right)\left(1-e^{E\tau }\right)}{E^{2}}dE \end{array}$$

where *E*_0_ sets the peak position, λ and σ are the Lorentzian and Gaussian widths, respectively. The function *g*(τ) encodes information about the electronic structure near the Fermi level through ρ­(ω) = *JDoS*(ω)/*JDoS*′(0), with *JDoS* denoting the joint DoS and the prime indicating an energy derivative. The dimensionless parameter α reflects the strength of the many-body interactions, i.e., the influence of the low-energy *JDoS* on the line shape.^77,79^ In Figure 2b we report ρ­(ω)/ω for the different materials, together with the reference DS ρ obtained by assuming a constant DoS. Since the *JDoS* measures the number of available electronic states at a given energy, the shape of ρ­(ω)/ω reflects the low-energy electronic structure near the Fermi level. For pristine 2H-TaS_2_, the Fermi energy lies near the maximum of the DoS, resulting in a decrease of ρ­(ω)/ω as one moves away from zero energy. In contrast, Fe_1/3_TaS_2_ shows Fe-induced states both above and below the Fermi level, which increase the number of available states away from *E*_*F*_, effectively opening additional channels for core-level excitations. Co_1/3_TaS_2_ exhibits an intermediate behavior, as Co states contribute significantly near the Fermi level while also enhancing the low-energy DoS, leading to a more modest variation of ρ­(ω)/ω with energy. From eq 1, it follows that the shape of ρ­(ω)/ω directly influences the core-level line shape, producing distinct asymmetric tails for the three compounds. This is illustrated in Figure 2c, where the core-level spectra are computed using eq 1 with α = 0.2.

**Figure 2:**
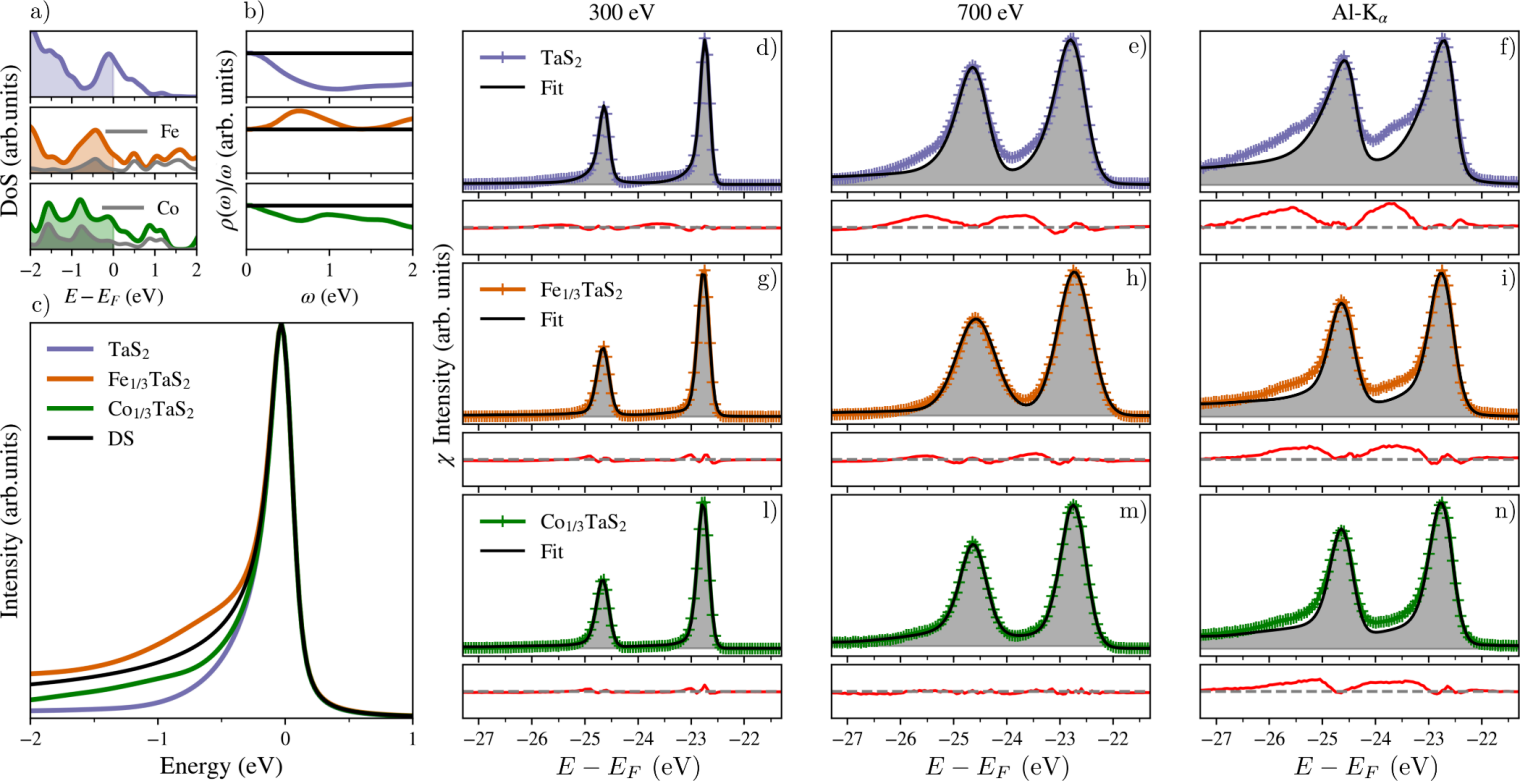
a) We report the Density of State (DoS) (in gray the projected DoS on Fe and Co atoms) and b) ρ­(ω)/ω (see main text) in 2H-TaS_2_, Fe_1/3_TaS_2_ and Co_1/3_TaS_2_, respectively, as calculated from DFT calculation. The black line in b) stand for ρ­(ω)/ω for a Doniach-Sunjach (DS) line shape corresponding to a flat DoS. In c) we show the material dependent core-level line shape definend by eq 1. d), e) and f) Photon-energy dependent Ta-4f core-level fit with the line shape defined in eq 1 for 2H-TaS_2_. In the bottom panels, the difference χ between the experimental data and the fitting function is reported. The vertical scale is kept identical for all χ plots to allow a direct comparison between the different measurements. The maximum and minimum ranges are determined by the largest χ signal, observed in Figure 2f. In g)-h)-i) and l)-m)-n) the same quantity for Fe_1/3_TaS_2_ and Co_1/3_TaS_2_ respectively.

Therefore, in Figure 2d–e–f, 2g–h–i, and 2l–m–n we report the results of the fitting procedure using the calculated line shape for 2H-TaS_2_, Fe_1/3_TaS_2_, and Co_1/3_TaS_2_, respectively, at different photon energies. The fitted values of the asymmetry parameter α are reported in Table 1. For all compounds, α shows an overall increase with photon energy, consistent with the progressive validity of the sudden approximation. Indeed, as discussed by Gadzuk and Šunjić,^83^ higher photoelectron kinetic energies lead to a faster creation of the core hole, driving the line shape toward the asymmetric limit. The larger fitted values of α from 2H-TaS_2_ to Co_1/3_TaS_2_ and Fe_1/3_TaS_2_ indicate an increasing density of states near the Fermi level and, consequently, a larger *JDoS*. This trend is consistent with the calculated DoS shown in Figure 2a, linking the asymmetry parameter α to the number of available electronic states at *E*_*F*_. A quantitative analysis of the residual χ, defined as the difference between the experimental spectra and the fitting function, further highlights a feature at ∼1 eV from the main peak in 2H-TaS_2_ (see Figure 2b–c–d), which cannot be reproduced by the core-level line shape modeling, even when including electronic structure effects. Applying the same analysis to Fe_1/3_TaS_2_ and Co_1/3_TaS_2_ reveals a marked suppression of this feature. In Fe_1/3_TaS_2_, a residual signal is still visible at *hν* = 700 eV and with the Al–K_α_ lamp source (see Figure 2e–f–g). In Co_1/3_TaS_2_, the feature is further suppressed and remains barely detectable, broad and weak, only in the Al–K_α_ spectra (see Figure 2h–i–l). Overall, the Ta-4*f* core-level analysis highlights a pronounced suppression of the extrinsic plasmon-loss signal upon intercalation (in Figures S3–S6 in SI we propose a fit with an asymmetric line shape as suggested in refs 63,84–86 ). At the same time, the spectra indicate efficient metallic screening and a modification of the low-energy electronic structure, consistent with a reduced DoS near the Fermi level.

**Table 1:** Asymmetry Parameter α for 2H-TaS_2_, Fe_1/3_TaS_2_, and Co_1/3_TaS_2_ Resulting from the Fitting Using Eq. 1 at Different Photon Energies h*ν* ^a^

	300 eV	700 eV	Al–K_α_
2H-TaS_2_	0.17	0.41	0.75
Fe_1/3_TaS_2_	0.04	0.03	0.12
Co_1/3_TaS_2_	0.10	0.13	0.21

a See Figure 2.

To further elucidate the microscopic origin of plasmon suppression and directly access its momentum dependence, we computed the electron energy-loss function (ELF) within linear-response theory using the random phase approximation (RPA) as implemented in GPAW^59–61^ (see SI for details). In pristine 2H-TaS_2_, the calculated plasmon dispersion reproduces the characteristic negative momentum dependence observed in TMDs,^15,18,36,37^ reflecting the presence of isolated metallic bands and a highly nontrivial low-energy screening environment (Figure 3a–b). In the SI (Section “ELF with CDW”), we also report the plasmon dispersion in the presence of the CDW distortion. The CDW has no qualitative effect on the plasmon dispersion, which remains negative. The only significant change is a small reduction of the peak intensity, which can be attributed to enhanced interband transitions associated with folded bands that acquire spectral weight,^34^ further demonstrating that the CDW does not qualitatively modify the plasmon dispersion.^18,36,37^ Upon intercalation, this behavior is qualitatively altered. Both Fe and Co strongly suppress the plasmon peak in the ELF (Figure 3c–f), with Co producing the most pronounced effect. This suppression is not merely a renormalization of the plasmon energy, but signals a breakdown of a well-defined collective mode. Microscopically, two concurrent mechanisms drive this evolution. First, intercalation reduces the bare plasma frequency, shifting the zeros of Re­(ϵ) to lower energies (see Figure 3e-f-g). Second, and more importantly, the intercalant-induced electronic states introduce a dense continuum of low-energy particle–hole excitations (Figure 1 and Figure 3e-f-g), which provide efficient decay channels for the plasmon. As a result, the collective excitation becomes strongly damped and progressively loses its coherence. This interpretation is directly confirmed by the calculated ELF: the plasmon mode of 2H-TaS_2_, which originates from the isolated metallic bands,^35^ evolves into a broad, overdamped feature in the intercalated compounds due to the enhanced phase space for electronic excitations. Crucially, this behavior is fundamentally different from conventional electron doping. In the latter case, increasing carrier concentration preserves the coherence of the plasmon and typically sharpens the peak while modifying its dispersion, as shown in Figure 3g–h and reported experimentally in ref.^87^ Our results therefore identify intercalation as a distinct route to control collective excitations: rather than tuning carrier density alone, it reshapes the low-energy electronic structure through orbital hybridization and band reconstruction, effectively transforming a well-defined plasmon into a strongly damped excitation. This establishes a direct link between chemical complexity and dynamical screening, and highlights intercalation as a powerful tool to engineer plasmonic responses in layered quantum materials.

**Figure 3:**
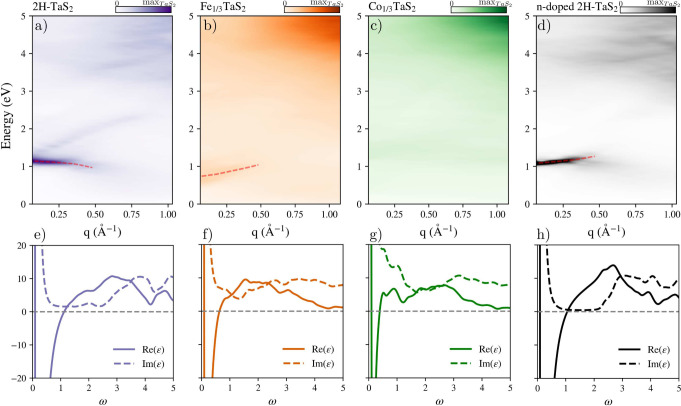
a)-b)-c)-d) Energy-loss function heat map as a function of energy and momentum for 2H-TaS_2_, Fe_1/3_TaS_2_, Co_1/3_TaS_2_ and electron doped 2H-TaS_2_ as calculated from DFT calculation. The heat map is calculated by linear interpolation of fixed momentum energy-loss function (see Methods for further details). In e)-f)-g)-h) we report real (solid line) and imaginary (dashed line) part of the dielectric function at *q* = 0 for all the discussed cases.

In conclusion, our combined spectroscopy and first-principles analysis demonstrates that Fe and Co intercalation provides a direct route to suppress and control the plasmon excitation characteristic of pristine 2H-TaS_2_. This quenching arises not from simple electron doping but from structural reconstruction, orbital hybridization, and enhanced low-energy absorption channels that disrupt coherent screening. First-principle calculations confirm the loss of a sharp plasmon mode and a substantial modification of the peculiar negative dispersion in TMDs. These results establish intercalation as a chemically controlled route to engineer dynamical screening in layered metals highlighting a general mechanism for manipulating collective excitations in van der Waals quantum materials.

## Supplementary Material



## Data Availability

All data that support the findings of this study are included within the article and Supporting Information.

## Notes

Experimental and theoretical methods; Plasma frequencies; ELF in the CDW phase; XPS data analysis (PDF)
