# Supplementary material for: Plasmon Engineering in Intercalated 2H-TaS2
Source: Nano Lett. 2026 Jul 10;26(28):9083–90. doi: 10.1021/acs.nanolett.6c01548 (PMC13397885; doi:10.1021/acs.nanolett.6c01548)
Supplement: Supplementary file 1 [file nl6c01548_si_001.pdf]

Supplemental material and supporting information for

Plasmon Engineering in Intercalated 2H-TaS_2

Luigi Camerano[a,b], Laura Martella [a], Lorenzo Battaglia [a], Federico Giannessi[a,b] Filippo Camilli [a] Polina M. Sheverdyaeva [c], Paolo Moras [c], Luca Lozzi [a], Luca Ottaviano[a,b], Gianni Profeta [a,b], Federico Bisti [a]

[a] Department of Physical and Chemical Sciences, University of L'Aquila, Via Vetoio 67100 L'Aquila, Italy
[b] CNR-SPIN L'Aquila, Via Vetoio, 67100 L'Aquila, Italy c/o Department of Physical and Chemical Sciences, University of L'Aquila, Via Vetoio, 67100 L'Aquila, Italy
[c] CNR-Istituto di Struttura della Materia (CNR-ISM), Strada Statale 14, km 163.5, 34149 Trieste, Italy

CONTENTS

I. Experimental methods 2

II. Theoretical simulations 2
A. ELF Computational details (GPAW) 3

III. Plasma frequencies and dielectric function 3

IV. ELF with CDW 4

V. Fermi level alignment 5

VI. XPS data analysis 6
A. Discussion on possible contamination 8

References 10

I. EXPERIMENTAL METHODS

The samples used in this study consist of commercially available crystals purchased from HQ Graphene.
Two independent sets of photoemission measurements were performed under different experimental conditions. Laboratory XPS measurements were acquired using a PHI 1257 spectrometer equipped with a monochromatized Al K_α source. The samples were cleaved in a load-lock chamber (base pressure $\sim 1 \times 10^{-7}$ mbar) and subsequently transferred to the analysis chamber, which operated at a base pressure of 1×10^{-9} mbar. Spectra were collected with a pass energy of 11.75 eV, corresponding to an overall experimental resolution of 0.25 eV, at room temperature. Under these conditions, a small surface-oxidation contribution could be detected in the Ta core-level spectra and was included in the fitting procedure discussed in the Supplementary Information.
LEED and photon-energy-dependent core-level photoemission spectroscopy measurements were instead performed at the VUV-Photoemission beamline (Elettra, Trieste). In this case, the samples were cleaved *in situ* and measured at $T = 20$ K under ultrahigh-vacuum conditions with a base pressure of 1×10^{-10} mbar. Owing to the cleaner surface preparation and improved vacuum conditions, no detectable oxidation-related spectral components were observed in these measurements.

II. THEORETICAL SIMULATIONS

Density functional theory calculations were performed using the Vienna ab-initio Simulation Package (VASP) [1, 2], using the generalized gradient approximation (GGA) in the Perdew-Burke-Ernzerhof (PBE) parametrization for the exchange-correlation functional [3], including SOC, thus capturing hidden-spin polarazion of TMDs as predicted and measured in Refs. [4–9] (see Fig. S1 for a visualization of the hidden spin polarization in 2H-TaS_2). Interactions between electrons and nuclei were described using the projector-augmented wave method. Energy thresholds for the self-consistent calculation was set to 10^{-6} eV and force threshold for geometry optimization 10^{-4} eV Å^{-1}. The Brillouin zone was sampled using an 8 × 8 × 4 Gamma-centered Monkhorst-Pack grid. To account for the on-site electron-electron correlation on localized Fe-d orbitals we used the GGA+U approach with an effective Hubbard term $U = 1.5$ eV. The 2H-TaS_2 lattice parameter are set to the experimental ones: $a = 3.31$ Å and $c = 12.07$ Å [10], while for the $Fe_{1/3}TaS_2$ $a = 5.737$ Å and $c = 12.28$ Å [11, 12] and $Co_{1/3}TaS_2$ $a = 5.725$ Å and $c = 11.878$ Å [13]. Due to the localization of Fe-d orbitals, different metastable phases can be stabilized for $Fe_{1/3}TaS_2$. To stabilize large orbital moment phase we used a mixing parameter $\alpha_{mix} = 0.22$ and the occupation matrix control as implemented in VASP [14]. We notice that this phase is indeed the ground state of the system by comparing the total energy. The bare plasma frequencies are calculated by sampling the BZ using an 12 × 12 × 5 Gamma-centered Monkhorst-Pack grid.

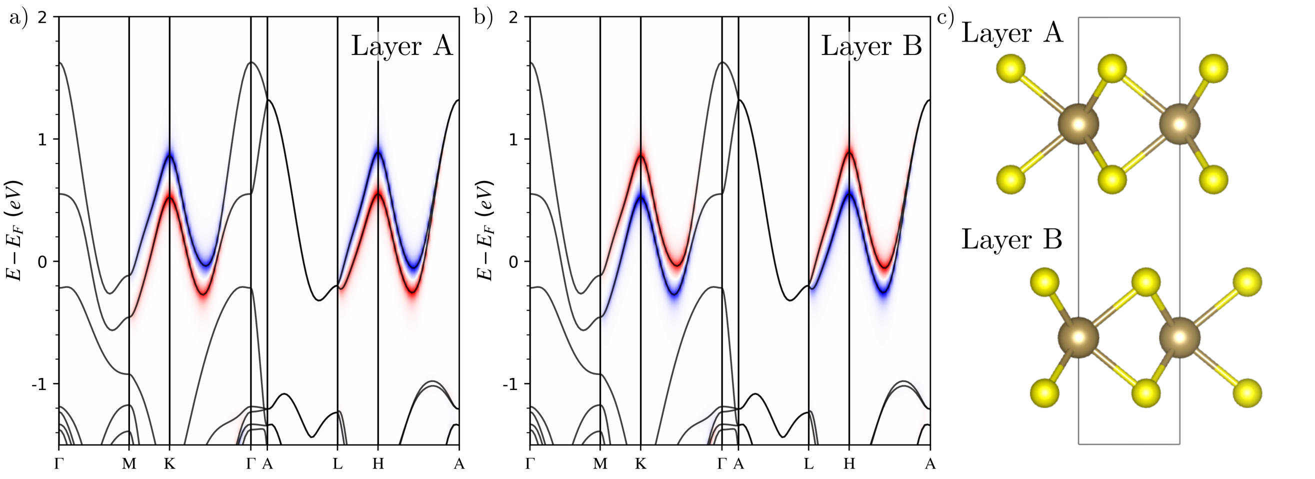

FIG. S1. Hidden spin-polarization in 2H-TaS_2. In panel (a) we report the spin-projected band structure on the layer A of the unit cell (see panel (c) for a visualization of the unit cell) while in (b) the projection on layer B. The colormap stems for $\langle S_z \rangle$.

A. ELF Computational details (GPAW)

To calculate the energy loss function (ELF), DFT calculations were performed using the projector augmented-wave (PAW) method as implemented by GPAW [15–17]. The exchange-correlation effects were treated using the PBE functional within the GGA approximation. Core-valence interactions were described using the PAW datasets supplied with GPAW. The experimentally reported crystal structures of TaS_2 and its Fe- and Co-intercalated derivatives were used without structural relaxation. For the self-consistent calculation of TaS_2, a plane-wave cutoff energy of 500 eV and a Monkhorst–Pack k-point mesh of 10 × 10 × 5 were employed together with a Fermi–Dirac smearing of 0.05 eV. For the Fe- and Co-intercalated systems, a cutoff energy of 600 eV and a 6 × 6 × 3 k-point mesh were used, together with a 0.2 eV Fermi-Dirac smearing. The convergence criteria and number of bands were kept at the default values implemented in GPAW. Spin-polarized calculations were performed for the intercalated systems. An initial magnetic moment of $2\,\mu_B$ was assigned to the Fe and Co atoms, finally converging to $3\,\mu_B$ for Fe and $1.3\,\mu_B$ for Co. For the Fe-intercalated system a ferromagnetic ordering was imposed while for Co-intercalated system an A-type antiferromagnetic configuration was imposed as the initial magnetic ordering. The final magnetic states were obtained self-consistently within the SCF procedure and used for subsequent calculations. For the intercalated systems, electronic convergence was also facilitated using the density mixing scheme MixerSum, with parameters $\beta = 0.1$, nmaxold = 5 and weight = 50.0). For the Fe-intercalated system, on-site Coulomb interactions were included within the DFT+U formalism with $U = 1.5$ eV applied to the Fe d states.
For the purposes of the present first-principles modelling, we have adopted an A-type antiferromagnetic configuration for $Co_{1/3}TaS_2$, which can be stabilized within the unit cell and provides a realistic and computationally tractable approximation. Importantly, this choice yields a magnetic moment on the Co site of approximately $\simeq 1.3\,\mu_B$, in very good agreement with experimental values reported from neutron scattering measurements (approximately $\simeq 1.28\,\mu_B$, Ref. [18]). Importantly, the main electronic structure features in this material are not determined by the magnetic configuration [19]. The electron-doped 2H-TaS_2 case is calculated by adding one electron in the unit-cell of 2H-TaS_2. Non-self-consistent calculations were next carried out using the converged ground-state density to increase the Brillouin zone sampling. The k-point meshes were refined to 30 × 30 × 15 for $TasS_2$ and 20 × 20 × 10 for the intercalated systems. The dielectric function and energy loss function were calculated within the linear response formalism using the random phase approximation (RPA) as implemented in GPAW. A local field effect cutoff of 100 eV was included in the response function calculation, together with a broadening parameter of $\eta = 25 \cdot 10^{-3}$ eV. In the case of the calculation of the response with CDW the local field effects were not included. The frequencies ranged from 0.01 to 5 eV. The macroscopic dielectric function was computed along the x-direction using the same parameters. The momentum-resolved ELF was evaluated along the Γ–M direction for momentum transfers ranging from 0.06, Å^{-1} to 1.07, Å^{-1}.

III. PLASMA FREQUENCIES AND DIELECTRIC FUNCTION

In this section we report the calculated bare plasma frequencies for the different compounds in Table S1 and the real $\mathrm{Re}(\epsilon)$ and imaginary $\mathrm{Im}(\epsilon)$ part of the dielectric function. We note the in-plane bare plasma frequency $\omega_{p,\parallel}$ decreases upon intercalation while the out-of-plane bare plasma frequency $\omega_{p,\perp}$ increases. This is pointing to an out-of-plane hybridization induced by intercalation as confirmed in ARPES [20]. The decreasing of in-plane plasma frequency directly influence the zero of the real part of the dielectric function $\mathrm{Re}(\epsilon(\omega))$ which determine the plasmon resonance condition as reported in Fig.3 of the main text. As discussed in the main manuscript, the suppression of the plasmon is due to the combined effect of a reduction of the bare plasma frequency and the introduction of additional low-energy states upon intercalation resulting in incresed $\mathrm{Im}(\epsilon(\omega))$ at low energy.

TABLE S1. In-plane (∥) and out-of-plane (⊥) bare plasma frequencies (ω_p) in eV for TaS_2, $Fe_{1/3}TaS_2$, and $Co_{1/3}TaS_2$.

	$\omega_{p,\parallel}$	$\omega_{p,\perp}$
TaS_2	3.24	0.28
$Fe_{1/3}TaS_2$	2.56	1.11
$Co_{1/3}TaS_2$	1.88	0.97

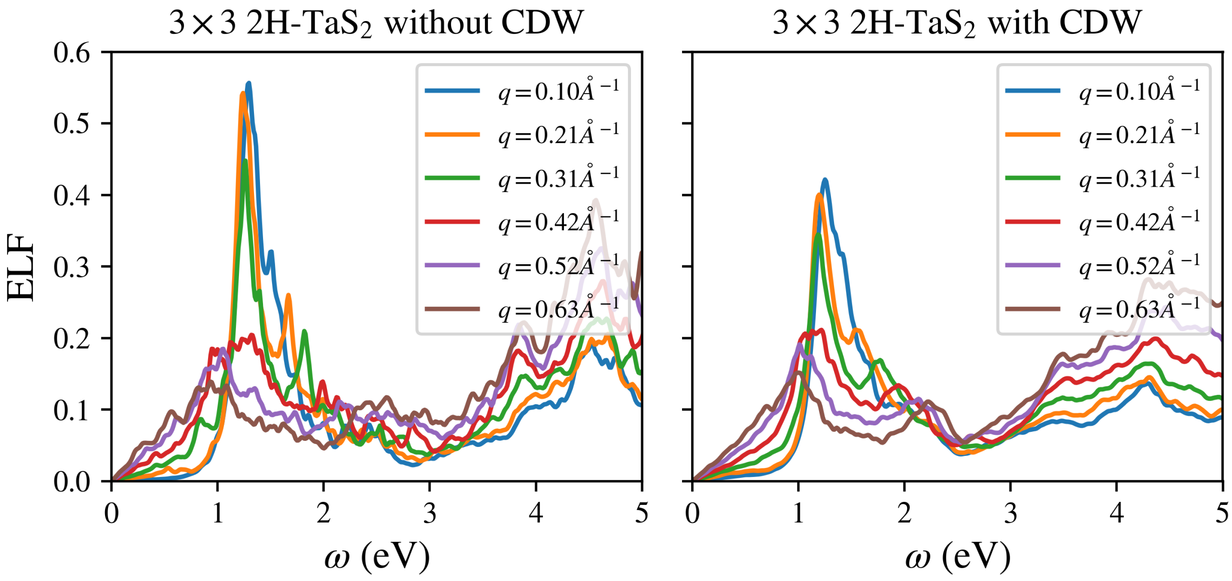

FIG. S2. a) ELF calculation in 3×3 supercell without CDW distortion and b) ELF calculation in 3×3 supercell with CDW distortion.

IV. ELF WITH CDW

In this section we report calculation of the ELF with and without CDW distortion. The CDW distortion is the same used in Ref. [21], that allowed us to compare the band structure with the ARPES measurement in 2H-TaS_2. In Fig. S2 we report the ELF in the 3×3 supercell in the absence (Fig. S2a) and presence (Fig. S2b) of the CDW distortion. The CDW has no qualitative effect on the plasmon dispersion, which remains negative. The only significant change is a small reduction of the peak intensity, which can be attributed to enhanced interband transitions associated to folded bands that acquire spectral weight [21]. This indicates that the plasmon is not suppressed neither its dispersion is modified, consistent with the metallic character of the system in the CDW phase (i.e., in the absence of a CDW induced full gap [21]).

V. FERMI LEVEL ALIGNMENT

In Fig. S3 we report the Fermi-edge fits obtained using a logistic function, while Fig. S4 shows the valence band, Ta-4f, and S-2p spectra for all investigated compounds, aligned to the corresponding fitted Fermi level. As discussed in the main text, Fig. S4a shows a doping of the valence band which is absent in the Ta-4f core level Fig. S4c, signaling efficient metallic screening.

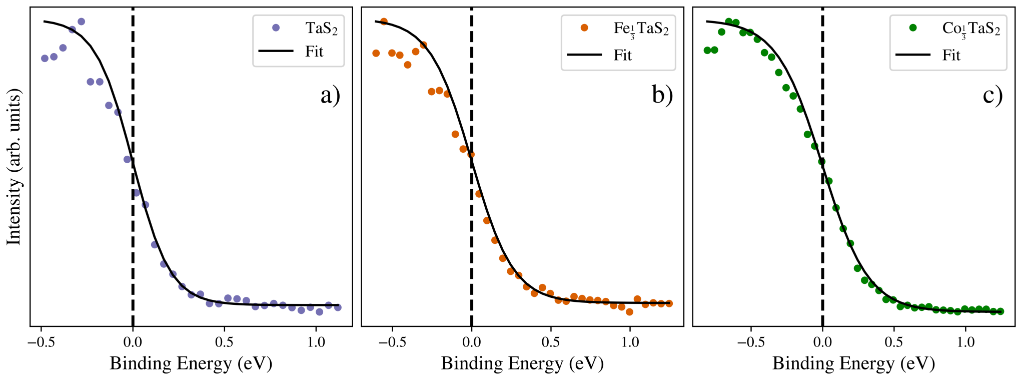

FIG. S3. Fitted Fermi level edge for 2H-TaS_2 (a), $Fe_{1/3}TaS_2$ (b) and $Co_{1/3}TaS_2$ (c).

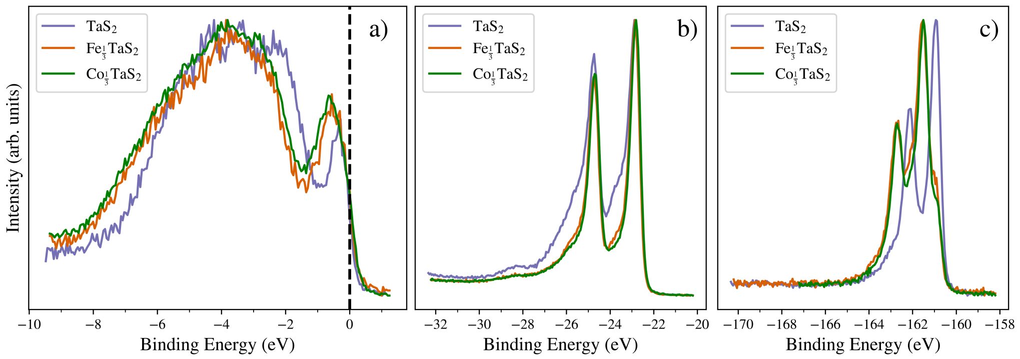

FIG. S4. Valence band (a), Ta-4f (b) and S-2p (c) core-level spectra acquired at room temperature using Al-K_α source ($h\nu$ = 1486.7 eV) for 2H-TaS_2, $Fe_{1/3}TaS_2$ and $Co_{1/3}TaS_2$. All spectra are referenced to the respective Fermi energies.

VI. XPS DATA ANALYSIS

In this section, we introduce a phenomenological lineshape that accounts for both JDoS effects and extrinsic losses in the core levels of 2H-TaS_2 and its intercalated counterparts. In Fig. S5, we demonstrate that a simple Voigt doublet is insufficient to capture the loss features and the asymmetry arising from the electronic JDoS. To address this, we adopt a skew normal distribution (SKND), which provides an improved description of the lineshape in these systems. The effectiveness of this approach is illustrated in Figs. S6 and S7, where the experimental spectra are accurately reproduced. Additionally, in Fig. S6 we identify an extra component with a main peak centered at 26 eV, which we attribute to the formation of Ta_2O_5 (Ta^{5+}) [22]. Finally, Fig. S8 shows the S-2p core-level spectra of $Co_{1/3}TaS_2$ as a function of the emission angle. The ratio between bulk and surface components, reported in Table S2, further supports the surface origin of the additional spectral feature.
The analytic expression of the SKND is:

$$N(E;\mu,\sigma,\alpha) = \frac{2\mathrm{A}}{\sigma}\,\phi\left(\frac{E-\mu}{\sigma}\right)\Phi\left(\alpha\cdot\frac{E-\mu}{\sigma}\right) \tag{1}$$

with:

- $\phi(E;\mu,\sigma) = \frac{1}{\sqrt{2\pi\sigma^2}}e^{-\frac{(E-\mu)^2}{2\sigma^2}}$ is the standard normal distribution,
- $\Phi(E;\mu,\sigma) = \frac{1}{2}\left[1+\mathrm{erf}\left(\frac{E-\mu}{\sqrt{2}\sigma}\right)\right]$ is the associated partition function,
- $\alpha \in \mathbb{R}$ is the asymmetry parameter.
- A is the amplitude.

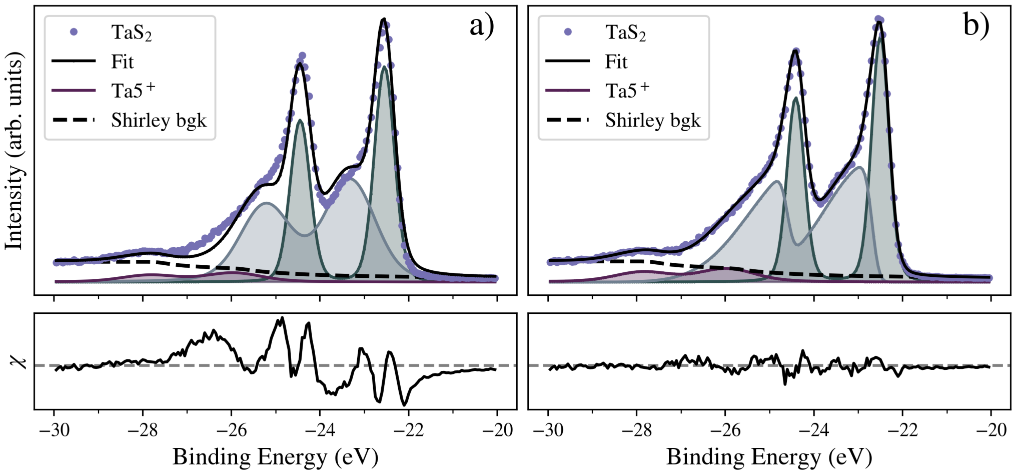

FIG. S5. Ta-4f core-level spectra of 2H-TaS_2 acquired at room temperature using an Al-K_α source ($h\nu$ = 1486.7 eV). Top panels a) and b) show the experimental data and corresponding fits, while the bottom panels display the residuals χ between the data and the fitting functions. The vertical scale is kept identical for all χ plots to allow a direct comparison between the different measurements. The maximum and minimum ranges are determined by the largest χ signal, observed in panel a). a) The fit consists of three Voigt doublets: the main emission, an additional feature accounting for the extrinsic plasmon, and a Ta^{5+} doublet due to surface oxidation. b) The fit utilizes Voigt functions for the main and oxidation doublets, and a Skew Normal Distribution (SKND) to describe the both the extrinsic plasmon and asymmetry of the peak due to electronic JDoS effects. In both panels, the black dashed line represents the Shirley background and all the doublets share the same ratio and peaks distance.

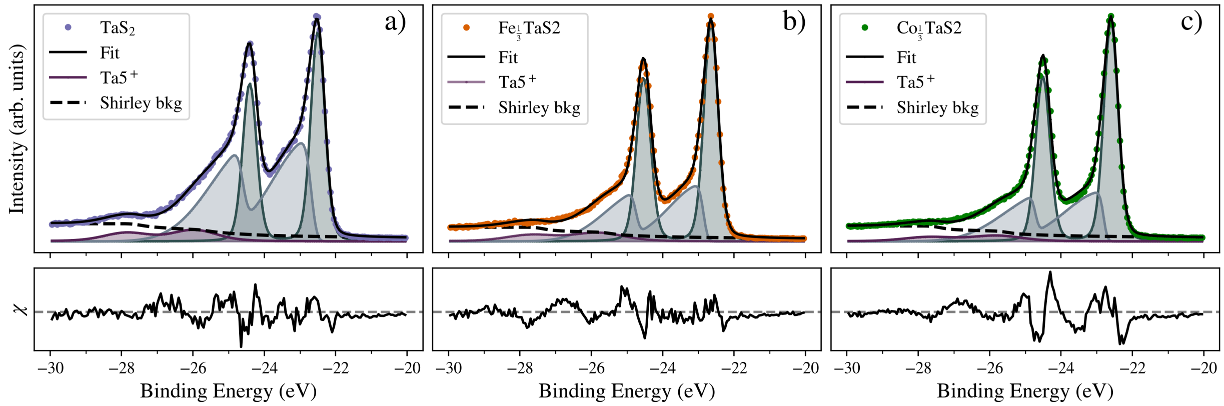

FIG. S6. Ta-4f core-level spectra of 2H-TaS_2 (a), $Fe_{1/3}TaS_2$ and $Co_{1/3}TaS_2$ acquired at room temperature using an Al-K_α source ($h\nu$ = 1486.7 eV). Top panels show the experimental data and corresponding fits, which uses SKND for the extrinsic plasmon. Bottom panels display the residuals χ between the data and the fitting functions. All plots share the same energy scale. The maximum and minimum ranges are determined by the largest χ signal, observed in panel a). The black dashed line represent the Shirley background. All the doublets share the same ratio and peaks distance.

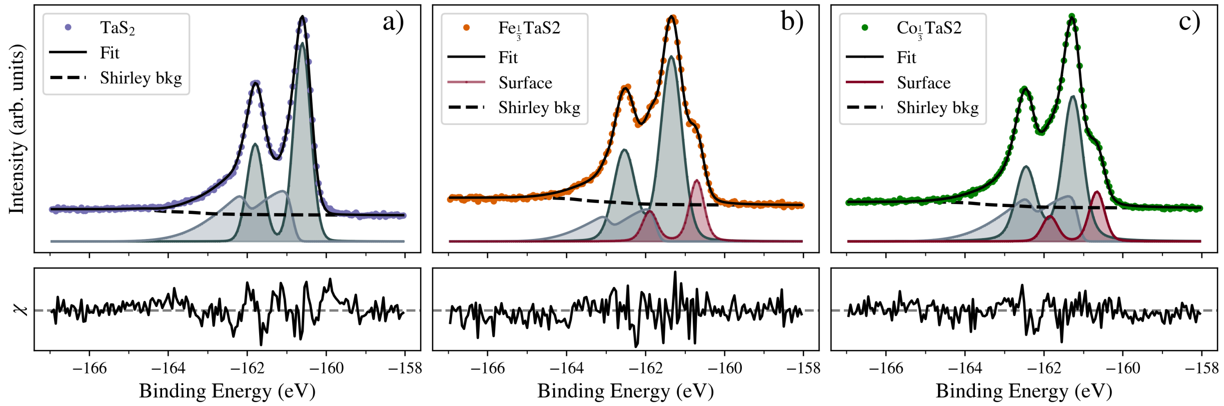

FIG. S7. S-2p core-level spectra acquired at room temperature using an Al-K$_\alpha$ source ($h\nu$ = 1486.7 eV). The bottom panels display the residuals χ between the data and the fitting functions The vertical scale is kept identical for all χ plots to allow a direct comparison between the different measurements. The maximum and minimum ranges are determined by the largest χ signal, observed in panel a). a) 2H-TaS_2 spectrum, the fit employs a Voigt doublet for the main emission and a SKND doublet for the extrinsic plasmon. b) 2H-$Fe_{1/3}TaS_2$ spectrum, the fit consists in 2 Voigt doublets for main emission and surface S, and a SKND doublet. c) Same quantities for 2H-$Co_{1/3}TaS_2$. All the doublets share the same ratio and peaks distance. The black dashed line represent the Shirley background. Bottom panels display the difference χ between the data and the fitting functions. All plots share the same energy scale.

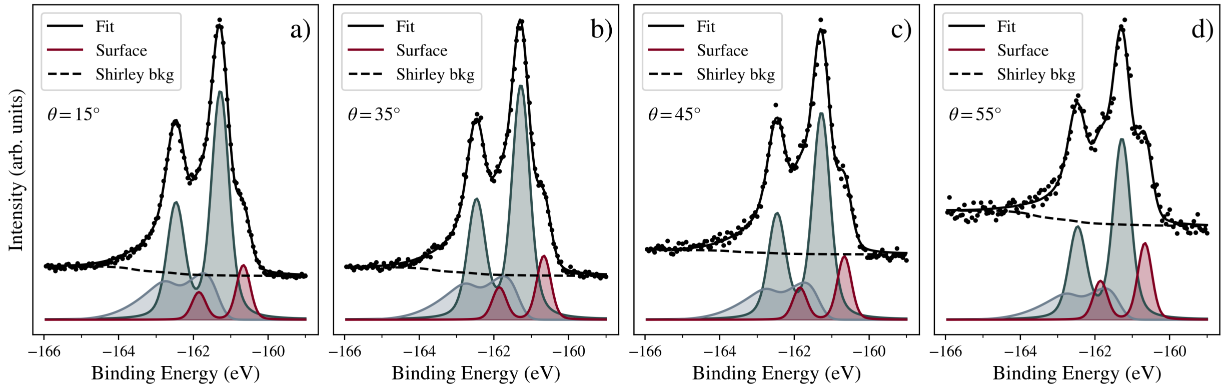

FIG. S8. S-2p core-level spectra of 2H-$Co_{1/3}TaS_2$ acquired at room temperature using an Al-K$_\alpha$ source ($h\nu$ = 1486.7 eV), varying the angle between the incident radiation and the sample. In each panel the fit consists in 2 Voigt doublets for main emission, surface S and a SKND doublet. The lineshape parameters are fixed to the values derived from the fit in Fig. S7 c), leaving only the peak intensities as free parameters. The black dashed line represent the Shirley background. All plots share the same energy scale.

TABLE S2. Ratio between the area of the main emission peak and the peak of surface S

	15°	35°	45°	55°
$A_{surface}/A_{main}$	0.239	0.273	0.307	0.424

A. Discussion on possible contamination

In Ref. [23], the Ta-4f core-level spectrum was modeled using multiple components, two of which were attributed to contamination effects, with the absence of plasmon-related losses ascribed to the low crystallinity of the samples. Our measurements are performed on high-quality single crystals cleaved *in situ* under ultra-high vacuum conditions, ensuring pristine and well-ordered surfaces. The resulting spectra, particularly those acquired at the synchrotron, exhibit sharp and well-defined Ta-4f and S-2p core-level features, indicative of a uniform chemical environment across

the sample. Moreover, contamination-related contributions are expected to be surface-sensitive and therefore enhanced at lower photon energies, where the probing depth is reduced. This behavior is not observed in our data. On the contrary, the ∼1 eV feature in the Ta-4f spectra increases in intensity with increasing photon energy, consistent with a bulk-sensitive origin. This trend follows the increase of the inelastic mean free path, which enhances the probability of photoelectrons undergoing inelastic scattering processes before escaping the sample, supporting its assignment to an extrinsic loss feature that are enanched with photon energy as is shown in Ref. [24] where Hard-X-rays XPS were used to study extrinsic plasmon excitation in aluminum. Further evidence against contamination effects is provided by the S-2p spectra of pristine 2H-TaS_2, which display a single, well-defined main component with no additional features across all measured photon energies. Indeed, possible sulfur oxidation states will appear at lower binding energy (in the range (-166,-170) eV) as reported in Ref. [25]. Additionally, no signatures of Ta oxides formation are observed in the Ta-4f lineshape in our synchrotron measurements, in contrast to laboratory XPS data where surface oxidation can be detected. Finally, the additional shoulder in S-2p core-level fitted with asymmetric lineshape in Fig. S7, that is more evident in 2H-TaS_2 spectra, further confirm the presence of losses in core-level spectra. Taken together, these observations demonstrate that the additional spectral weight observed in the core levels originates from electronic excitations of the material, manifesting as extrinsic plasmon-loss processes during photoemission, rather than from contamination or sample inhomogeneity.

[1] G. Kresse and J. Hafner, "Ab initio molecular dynamics for liquid metals," Phys. Rev. B **47**, 558–561 (1993).

[2] G. Kresse and D. Joubert, "From ultrasoft pseudopotentials to the projector augmented-wave method," Phys. Rev. B **59**, 1758–1775 (1999).

[3] John P. Perdew, Kieron Burke, and Matthias Ernzerhof, "Generalized gradient approximation made simple," Phys. Rev. Lett. **77**, 3865–3868 (1996).

[4] Xiuwen Zhang, Qihang Liu, Jun-Wei Luo, Arthur J. Freeman, and Alex Zunger, "Hidden spin polarization in inversion-symmetric bulk crystals," Nature Physics **10**, 387–393 (2014).

[5] L. Bawden, S. P. Cooil, F. Mazzola, J. M. Riley, L. J. Collins-McIntyre, V. Sunko, K. W. B. Hunvik, M. Leandersson, C. M. Polley, T. Balasubramanian, T. K. Kim, M. Hoesch, J. W. Wells, G. Balakrishnan, M. S. Bahramy, and P. D. C. King, "Spin–valley locking in the normal state of a transition-metal dichalcogenide superconductor," Nature Communications **7** (2016), 10.1038/ncomms11711.

[6] J. M. Riley, F. Mazzola, M. Dendzik, M. Michiardi, T. Takayama, L. Bawden, C. Granerød, M. Leandersson, T. Balasubramanian, M. Hoesch, T. K. Kim, H. Takagi, W. Meevasana, Ph. Hofmann, M. S. Bahramy, J. W. Wells, and P. D. C. King, "Direct observation of spin-polarized bulk bands in an inversion-symmetric semiconductor," Nature Physics **10**, 835–839 (2014).

[7] Linding Yuan, Qihang Liu, Xiuwen Zhang, Jun-Wei Luo, Shu-Shen Li, and Alex Zunger, "Uncovering and tailoring hidden rashba spin–orbit splitting in centrosymmetric crystals," Nature Communications **10** (2019), 10.1038/s41467-019-08836-4.

[8] Avior Almoalem, Roni Gofman, Yuval Nitzav, Ilay Mangel, Irena Feldman, Jahyun Koo, Federico Mazzola, Jun Fujii, Ivana Vobornik, J. S´anchez-Barriga, Oliver J. Clark, Nicholas Clark Plumb, Ming Shi, Binghai Yan, and Amit Kanigel, "Charge transfer and spin-valley locking in 4Hb-TaS_2," npj Quantum Materials **9** (2024), 10.1038/s41535-024-00646-2.

[9] Wenxin Li, Jonathan T. Reichanadter, Shan Wu, Ji Seop Oh, Rourav Basak, Shannon C. Haley, Siqi Wang, Joshua E. Chaparro Mata, Elio Vescovo, Donghui Lu, Makoto Hashimoto, Christoph Klewe, Suchismita Sarker, Jessica L. McChesney, Alex Frañó, James G. Analytis, Robert J. Birgeneau, Jeffrey B. Neaton, and Yu He, "Electronic origin of delicate antiferromagnetism in $fe_x nbs_2$," Phys. Rev. Lett. **136**, 216503 (2026).

[10] Yiwei Hu, Qiaoyan Hao, Baichuan Zhu, Biao Li, Zhan Gao, Yan Wang, and Kaibin Tang, "Toward exploring the structure of monolayer to few-layer TaS_2 by efficient ultrasound-free exfoliation," Nanoscale Research Letters **13** (2018), 10.1186/s11671-018-2439-z.

[11] J Dijkstra, P J Zijlema, C F van Bruggen, C Haas, and R A de Groot, "Band-structure calculations of $Fe_{1/3}TaS_2$ and $Mn_{1/3}TaS_2$, and transport and magnetic properties of $Fe_{0.28}TaS_2$," *JournalofPhysics : CondensedMatter* **1**, 6363–6379(1989).

[12] Sebastian Mangelsen, Julian Hansen, Peter Adler, Walter Schnelle, Wolfgang Bensch, Sergiy Mankovsky, Svitlana Polesya, and Hubert Ebert, "Large anomalous Hall effect and slow relaxation of the magnetization in $Fe_{1/3}TaS_2$," The Journal of Physical Chemistry C **124**, 24984–24994 (2020).

[13] Pyeongjae Park, Woonghee Cho, Chaebin Kim, Yeochan An, Maxim Avdeev, Kazuki Iida, Ryoichi Kajimoto, and Je-Geun Park, "Composition dependence of bulk properties in the co-intercalated transition metal dichalcogenide $co_{1/3}Tas_2$," Phys. Rev. B **109**, L060403 (2024).

[14] Jeremy P. Allen and Graeme W. Watson, "Occupation matrix control of d- and f-electron localisations using dft + u," Phys. Chem. Chem. Phys. **16**, 21016–21031 (2014).

[15] Jens Jørgen Mortensen, Ask Hjorth Larsen, Mikael Kuisma, Aleksei V. Ivanov, Alireza Taghizadeh, Andrew Peterson, Anubhab Haldar, Asmus Ougaard Dohn, Christian Schäfer, Elvar Örn Jónsson, Eric D. Hermes, Fredrik Andreas Nilsson, Georg Kastlunger, Gianluca Levi, Hannes Jónsson, Hannu Häkkinen, Jakub Fojt, Jiban Kangsabanik, Joachim Sødequist, Jouko Lehtomäki, Julian Heske, Jussi Enkovaara, Kirsten Trøstrup Winther, Marcin Dulak, Marko M. Melander, Martin Ovesen, Martti Louhivuori, Michael Walter, Morten Gjerding, Olga Lopez-Acevedo, Paul Erhart, Robert Warmbier, Rolf Würdemann, Sami Kaappa, Simone Latini, Tara Maria Boland, Thomas Bligaard, Thorbjørn Skovhus, Toma Susi, Tristan Maxson, Tuomas Rossi, Xi Chen, Yorick Leonard A. Schmerwitz, Jakob Schiøtz, Thomas Olsen, Karsten Wedel Jacobsen, and Kristian Sommer Thygesen, "Gpaw: An open python package for electronic structure calculations," The Journal of Chemical Physics **160** (2024), 10.1063/5.0182685.

[16] J. J. Mortensen, L. B. Hansen, and K. W. Jacobsen, "Real-space grid implementation of the projector augmented wave method," Phys. Rev. B **71**, 035109 (2005).

[17] J Enkovaara, C Rostgaard, J J Mortensen, J Chen, M Dułak, L Ferrighi, J Gavnholt, C Glinsvad, V Haikola, H A Hansen, H H Kristoffersen, M Kuisma, A H Larsen, L Lehtovaara, M Ljungberg, O Lopez-Acevedo, P G Moses, J Ojanen, T Olsen, V Petzold, N A Romero, J Stausholm-Møller, M Strange, G A Tritsaris, M Vanin, M Walter, B Hammer, H Häkkinen, G K H Madsen, R M Nieminen, J K Nørskov, M Puska, T T Rantala, J Schiøtz, K S Thygesen, and K W Jacobsen, "Electronic structure calculations with gpaw: a real-space implementation of the projector augmented-wave method," Journal of Physics: Condensed Matter **22**, 253202 (2010).

[18] Pyeongjae Park, Woonghee Cho, Chaebin Kim, Yeochan An, Yoon-Gu Kang, Maxim Avdeev, Romain Sibille, Kazuki Iida, Ryoichi Kajimoto, Ki Hoon Lee, Woori Ju, En-Jin Cho, Han-Jin Noh, Myung Joon Han, Shang-Shun Zhang, Cristian D. Batista, and Je-Geun Park, "Tetrahedral triple-Q magnetic ordering and large spontaneous Hall conductivity in the metallic triangular antiferromagnet $Co_{1/3}TaS_2$," Nature Communications **14** (2023), 10.1038/s41467-023-43853-4.

[19] Wojciech Sas, Yuki Utsumi Boucher, Seyed Ashkan Moghadam Ziabari, Gaurav Pransu, Trpimir Ivšić, Ivana Vobornik, Jun Fujii, Naveen Singh Dhami, Bruno Gudac, Mario Novak, László Forró, Neven Barišić, Ivo Batistić, and Petar Popčević,

"Origin of a shallow electron pocket: -band in $co_{1/3}tas_2$ studied by angle-resolved photoemission spectroscopy," (2026), 10.48550/ARXIV.2602.15588.

[20] Luigi Camerano, Emanuel A. Martínez, Victor Porée, Laura Martella, Dario Mastrippolito, Debora Pierucci, Franco D'Orazio, Polina M. Sheverdyaeva, Paolo Moras, Enrico Della Valle, Tianlun Yu, Moritz Hoesch, Craig M. Polley, Thiagarajan Balasubramanian, Alessandro Nicolaou, Luca Ottaviano, Vladimir N. Strocov, Gianni Profeta, and Federico Bisti, "Emergent 3d fermiology and magnetism in an intercalated van der waals system," (2026).

[21] Luigi Camerano, Dario Mastrippolito, Debora Pierucci, Ji Dai, Massimo Tallarida, Luca Ottaviano, Gianni Profeta, and Federico Bisti, "Darkness in interlayer and charge density wave states of 2H-TaS_2," Phys. Rev. B **111**, L121112 (2025).

[22] Maxim V. Ivanov, Timofey V. Perevalov, Vladimir S. Aliev, Vladimir A. Gritsenko, and Vasily V. Kaichev, "Electronic structure of -ta2o5 with oxygen vacancy: ab initio calculations and comparison with experiment," Journal of Applied Physics **110**, 024115 (2011), https://pubs.aip.org/aip/jap/article-pdf/doi/10.1063/1.3606416/15078473/024115_1*online.pdf*.

[23] Charalampos Drivas, C. Richard A. Catlow, Mark A. Isaacs, and Georgios Kyriakou, "Considerations on the xps analysis of 2h-tas2(0001)," Surface and Interface Analysis **57**, 802–808 (2025).

[24] Mohammad Balal, Shuvam Sarkar, Pramod Bhakuni, Andrei Gloskovskii, Aparna Chakrabarti, and Sudipta Roy Barman, "Intrinsic and extrinsic plasmons in the hard x-ray photoelectron spectra of nearly free electron metals," Phys. Rev. B **109**, 205419 (2024).

[25] S Siow Kim, Leanne Britcher, Sunil Kumar, and Hans J Griesser, "Xps study of sulfur and phosphorus compounds with different oxidation states," Sains Malaysiana **47**, 1913–1922 (2018).
